# Mental Health Status and Its Influencing Factors: The Case of Nurses Working in COVID-19 Hospitals in South Korea

**DOI:** 10.3390/ijerph18126531

**Published:** 2021-06-17

**Authors:** Min-Young Kim, Yun-Yi Yang

**Affiliations:** 1 Department of Nursing, Ulsan University, Ulsan 44610, Korea; minyoung1026@gmail.com; 2 Department of Nursing, Healthcare Science & Human Ecology, Dong-Eui University, Busan 47340, Korea

**Keywords:** COVID-19, nurses, mental health status, South Korea, anxiety, depression, stress

## Abstract

The mental health of nurses participating in patient care is under threat amid the coronavirus disease 2019 (COVID-19) pandemic. This study aimed to identify the mental health status (depression, anxiety, and stress) and its influencing factors on nurses who provided patient care at a specialized hospital for COVID-19 in South Korea. Of the 180 nurses who participated in this study, 30.6% had moderate or higher levels of depression, 41% had moderate or higher anxiety levels, and 19.4% had moderate or higher stress levels. In this study, stigma influenced nurses’ mental health, such that the higher the stigma, the higher the nurses’ depression, anxiety, and stress. Depression was higher in female nurses than in male nurses, and stress was higher in charge nurses than nurses in other job positions. Therefore, a management program should be designed to improve the mental health of nurses during the current pandemic. In particular, a solution to reduce stigma is required, and the mental health of female nurses and nurses in leadership roles requires special attention.

## 1. Introduction

Coronavirus disease 2019 (COVID-19)—where the first cases were identified in Wuhan, China, in December 2019—is a respiratory syndrome caused by the SARS-CoV-2 virus that has now developed into a pandemic. Since the first outbreak on January 20, 2020, there have been 88,120 confirmed cases of COVID-19 in South Korea (as of 24 February 2021). The first specialized hospital for COVID-19 in South Korea was designated in Daegu on 18 February 2020, to cope with the explosive increase in the number of patients after the first confirmed case. The Keimyung University Daegu Dongsan Hospital, appointed as a specialized hospital for COVID-19, is the largest in the region. On 21 February 2020, all inpatients were discharged from the hospital, and the main building and entire research building were separated into cohort quarantine areas, where 385 beds were set up to treat COVID-19 patients.

Globally, healthcare workers infected with COVID-19 account for 4.4–11.0% of people infected with COVID-19 [1], and 22.8–23.2% of healthcare workers are at risk of developing mental health problems [2,3]. Compared to other healthcare workers, nurses are at higher risk of mental health repercussions as they provide medical services through direct contact with patients [3]. Studies in other countries have shown that nurses have developed high levels of depression and anxiety during the COVID-19 pandemic [3,4]. Therefore, it is necessary to develop solutions by identifying the mental health status and its influencing factors in nurses caring for COVID-19 patients.

Systematic reviews have identified factors affecting the mental health status of healthcare workers providing face-to-face care to patients. These factors included general characteristics such as gender, working in areas with a high risk of COVID-19 infection, medical history, distress caused by traumatic events, post-traumatic stress disorder, and social support [3,4]. Further, the stigma experienced by healthcare workers during a pandemic can pose a threat to their mental health [5]. In the current study, stigma refers to a situation where, in the context of an infectious disease, a person at high risk of infection is negatively evaluated by society, and therefore, experiences social exclusion, rejection, criticism, or devaluation. However, in South Korea, there are currently no studies on mental health status and its influencing factors in nurses caring for patients with COVID-19.

Therefore, this study aimed to investigate the mental health status (depression, anxiety, and stress) of nurses who participated in patient care at a specialized hospital for COVID-19 in South Korea. It also aimed to provide basic data for developing intervention programs and solutions to improve nurses’ mental health status during disease outbreaks by identifying its influencing factors.

## 2. Materials and Methods

### 2.1. Study Design

A descriptive survey was conducted to identify front-line nurses’ mental health status and determine the influencing factors thereof. A total of 45 items were used, which were structured (multiple choice questions) using a survey.

### 2.2. Setting and Participants

The criteria for the selection of participants included nurses who participated in patient care at the hospital. Nurses who were undergoing mental health-related treatments or taking medication related to depression, anxiety, and sleep were excluded.

The sample size required for this study was calculated using G*Power 3.12. To maintain a significance level of 0.05, an effect size of 1.5, and power of 0.80, the minimum required sample size was calculated to be 146. Therefore, 180 participants were recruited considering a dropout rate of 20%.

### 2.3. Research Tools

#### 2.3.1. General Characteristics

The participants’ general characteristics measured in this study included age, gender, marital status, whether they lived with their children, type of residence, job position, level of education, department, and nursing experience in treating patients with infectious diseases.

#### 2.3.2. Mental Health Status

The Korean version of the Depression Anxiety Stress Scales (DASS-21) developed by Henry and Crawford [6], and translated by Lee et al. [7] (Korean version of the Depression Anxiety Stress Scales: K-DASS-21), was used to assess mental health status. The K-DASS-21 is a measuring tool available for general use, and the Korean version was downloaded from the DASS website. Depression, anxiety, and stress are sub-areas of the K-DASS-21, with seven questions per area. A higher score indicates higher severity of depression, anxiety, and stress. To determine the level of depression, anxiety, and stress in each sub-area, the score of each sub-area is multiplied by two. A depression score of 0 to 9 points indicates a normal level of depression, a score of 10 to 13 points indicates mild depression, and a score of 14 or more indicates moderate to severe depression. An anxiety score of 0 to 7 points indicates a normal level of anxiety, a score of 8 to 9 points indicates mild anxiety, and a score of 10 or more points indicates moderate to severe anxiety. A stress score of 0 to 14 points indicates a normal level of stress, a score of 15 to 18 points indicates mild stress, and a score of 19 or more points indicates moderate to severe stress. When the tool was developed, Cronbach’s α was 0.88 for depression, 0.82 for anxiety, and 0.90 for stress; for the current study, Cronbach’s α was 0.89 for depression, 0.87 for anxiety, and 0.90 for stress.

#### 2.3.3. Stigma

Stigma is a personal experience of social exclusion, rejection, criticism, or devaluation caused by anticipating negative evaluations from society [8]. With the permission of the tool’s developers, Park et al. [9], the phrase “COVID-19” was inserted in the stigma scale used in this study for healthcare workers associated with the ongoing pandemic. This scale is composed of 16 questions rated on a five-point Likert scale. A higher score indicates a higher level of stigma. The tool’s Cronbach’s α at the time of development was 0.94 and 0.92 in this study.

### 2.4. Data Collection

Data collection was conducted over five days from 10 to 15 June 2020 (four months after the COVID-19 pandemic began) after receiving approval (DIRB-20205-HR-R-07) from the Institutional Review Board (IRB) of the University of Dong-Eui. The researchers collected data directly from nurses at a resting area shared by all healthcare workers in the uninfected annex of Keimyung University Dongsan Hospital, Daegu. After sufficient explanations of the study’s purpose, necessity, and procedure were provided, the researchers surveyed those who agreed to participate. The researchers collected the questionnaires and checked them for missing data; questionnaires were only given to those who agreed to complete them, and all the questionnaires that were handed out were completed in full, resulting in a response rate of 100%.

### 2.5. Data Analysis and Statistics

Using the SPSS/WIN 22.0 program, the data were analyzed as follows.

To determine the general characteristics of the participants’ mental health status (depression, anxiety, and stress) and stigma, the frequency, percentages, means, and standard deviations were calculated. According to the participants’ general characteristics, the differences in mental health status were analyzed using an independent t-test and one-way analysis of variance (ANOVA). The variables analyzed by ANOVA satisfied equal variance, and a post-test was performed using the Scheffe method. The relationship between the participants’ mental health status and stigma was analyzed using Pearson’s correlation analysis. The factors influencing the participants’ mental health status were analyzed using a stepwise multiple linear regression.

## 3. Results

### 3.1. Mental Health Status of Participants

Regarding the participants’ depression levels, 111 (61.7%) nurses did not have depression, 14 (7.8%) had mild depression, and 55 (30.6%) had moderate to severe depression; the average score was 9.32 ± 8.70 points, indicating a normal level of depression. Regarding anxiety levels, 96 (53.3%) participants did not have anxiety, 10 (5.6%) had mild anxiety, and 74 (41.1%) had moderate to severe anxiety; the average score was 8.70 ± 8.42 points, indicating a mild level of anxiety. Regarding stress levels, 126 (70.0%) participants did not experience stress, 19 (10.6%) were under mild stress, and 35 (19.4%) were under moderate to severe stress; the average score was 10.84 ± 9.57 points, indicating a normal level of stress. These results are shown in Table 1.

### 3.2. Differences in Mental Health Status According to General Characteristics of Participants

Among the participants’ general characteristics, the average age was 31.5 ± 8.60 years; 147 (81.7%) participants were less than 40 years, and 33 (18.3%) were aged 40 years or above. There were significant differences found in the depression (t = −3.29, *p* = 0.001), anxiety (t = −3.21, *p* = 0.002), and stress (t = −3.58, *p* < 0.001) levels of the participants (Table 2) according to age. Regarding gender, there were 10 (5.6%) men and 170 (94.4%) women. There were significant differences in the depression (z = −3.74, *p* < 0.001), anxiety (z = −3.10, *p* = 0.002), and stress (z = −2.68, *p* = 0.007) levels according to gender. Regarding marital status, 49 (27.2%) participants were married, and 31 (72.8%) were unmarried; there were no significant differences in the depression, anxiety, and stress levels according to marital status. Of the participants, 38 (21.1%) lived with children, while 142 (78.9%) did not, indicating significant differences in depression (t = −3.19, *p* = 0.001), anxiety (t = −3.14, *p* = 0.002), and stress (t = −3.53, *p* < 0.001). At the time of data collection, 70 (38.9%) participants lived in their own homes, and 110 (61.1%) lived in other places, with no significant differences in depression, anxiety, and stress according to residential status (Table 2).

Regarding the participants’ job positions, 156 (86.7%) were nurses, 13 (7.2%) were charge nurses, and 11 (6.1%) were head nurses or higher; the results revealed significant differences in depression (F = 6.60, *p =* 0.003), anxiety (F = 4.97, *p* = 0.008), and stress (F = 8.19, *p* < 0.001) according to the job position. Regarding the levels of education, 155 (86.1%) participants had a bachelor’s degree, 23 (12.8%) had a master’s degree, and 2 (1.1%) had a doctoral degree. Regarding the different departments represented in this sample, 95 (52.8%) participants were working in the COVID-19 ward, 72 (40.0%) in the intensive care unit, and 13 (7.2%) in the infection control administration department. Thirteen (7.2%) participants had experience working at an infectious disease-specialized hospital, and 167 (92.8%) were not. There were no significant differences in depression, anxiety, and stress according to the levels of education, department, or experience of working at a specialized hospital for infectious diseases (Table 2).

After examining the differences in depression, anxiety, and stress according to the participants’ general characteristics, it was found that the levels of depression, anxiety, and stress were higher in those aged 40 or above, in women, in those living with children, and in charge nurses. The average stigma score was 38.94 points (±12.37), and it had a significant positive correlation with depression (r = 0.42, *p* < 0.001), anxiety (r = 0.46, *p* < 0.001), and stress (r = 0.44, *p* < 0.001) (Table 2).

### 3.3. Factors Influencing the Mental Health Status of Participants

To identify the factors affecting the participants’ mental health status, a stepwise multiple linear regression was conducted. Age (reference group: 40 or above), gender (reference group: female), whether they lived with their children (reference group: those living with children), and job position (reference group: charge nurses) were entered as the variables.

Prior to performing multiple regression analysis with depression, anxiety, and stress as dependent variables, the normal P-P and residual scatter plots were examined to verify the basic assumptions of linearity, normality, independence, and homoscedasticity of error terms. In the normal P-P plot, there was no specific pattern between the two axes, which showed linearity, and the distribution of residuals was random in the scatter plot, which was distributed vertically around 0 to satisfy normality and homoscedasticity. The independence of observations in the multiple regression analysis was confirmed with depression, anxiety, and stress as the dependent variables.

The regression model in which depression was the dependent variable explained depression by 26% (F = 33.54, *p* < 0.001) (Table 3). The higher the stigma score (β = 0.41, *p* < 0.001), the higher the depression in women (β = 0.14, *p* = 0.036). The regression model in which anxiety was the dependent variable explained anxiety by 36% (F = 34.54, *p* < 0.001) (Table 4). The higher the stigma score (β = 0.46, *p* < 0.001), the higher the anxiety in participants. The regression model in which stress was the dependent variable explained 33% of the stress in participants (F = 30.85, *p* < 0.001) (Table 5). The higher the stigma score (β = 0.40, *p* < *0*.001), the higher the stress in charge nurses (β = 0.17, *p* = 0.014) when compared to nurses in other positions.

## 4. Discussion

This study was conducted to explore the mental health status (depression, anxiety, and stress) of nurses in a COVID-19-specialized hospital and to identify the factors affecting their mental health status. In this study, the average depression score of nurses working at the hospital was 9.32 points, which was within the normal range. The anxiety score was 8.70 points, indicating mild anxiety, and the stress score was 10.84 points, indicating a normal level of stress. This is similar to the results of previous studies in other countries, reporting a normal level of depression, mild anxiety, and a normal level of stress during the current COVID-19 pandemic [10,11]. Thus, it can be interpreted that similar levels of depression, anxiety, and stress have been experienced by nurses globally during this pandemic. However, 55 participants (30.6%) in this study had moderate to severe levels of depression. This is higher than those reported among nurses in China (0.6%) [10], and in the general public of South Korea (20.7%) [12].

Additionally, in this study, 74 participants (41.1%) had moderate to severe anxiety, which was higher than that reported among nurses in China (18.8%) [9], and in the general public of South Korea (22.4%) [12]. Furthermore, among the nurses in this study, 35 (19.4%) were under moderate to severe stress, which was higher than nurses in China (0%), and in the general public of South Korea (13.6%). One month after the COVID-19 outbreak, nurses’ depression, anxiety, and stress levels gradually declined, showing a pattern of adaptation to the situation and stabilization [13]. Since the data in this study were collected four months after the pandemic began, the mental health status of nurses was expected to have stabilized; however, many of the nurses in this study had depression, anxiety, and stress above the moderate level. Healthcare workers exposed to persistent depression, anxiety, and stress during a pandemic are at high risk of developing aftereffects such as post-traumatic stress syndrome [14]. Therefore, active investigation and interventions for the mental health of nurses with high levels of anxiety, depression, and stress are required.

Stigma was a significant factor affecting depression, anxiety, and stress among the nurses in this study. This result is similar to those of previous studies that identified stigma as a significant factor influencing the mental health status of South Korean hospital nurses during the Middle East respiratory syndrome (MERS) outbreak in South Korea [9]. This result is also consistent with those of a previous study that identified stigma as a significant influencing factor of stress in doctors [15] and dialysis staff [16] during the COVID-19 pandemic. In particular, the average stigma score of nurses in this study was 38.94 points, which was higher than that of South Korean nurses during the MERS outbreak, at 24.60 points [9]. The average score of doctors in other countries during the COVID-19 pandemic was 28.26 points [15], and that of the dialysis staff was 25.33 points [16]. The stigma faced by the nurses at Keimyung University Dongsan Hospital is particularly high compared to other countries and medical staff. As the removal/reduction of stigma requires legal and ethical measures and psychological support, strong support from national and local communities is required [17,18] to curb the stigma faced by nurses in COVID-19-specialized hospitals.

In this study, regarding gender, being a woman was a factor that significantly influenced depression. This is similar to the results of previous studies in which being female was identified as a factor influencing depression in healthcare workers [19] and the general public [20] in other countries during the COVID-19 pandemic. In addition, it is similar to the results of a previous study [13] wherein female nurses had significantly higher depression scores than male nurses. Being a charge nurse was also a factor influencing stress in nurses at the hospital. A head nurse manages the nursing unit, and a charge nurse is a registered nurse who oversees a unit during their shift [21]. The charge nurse’s duties include direct nursing and administrative work, and the scope of the job is often unclear [22].

This abovementioned result is inconsistent with Sun et al.’s [10], who reported that being a charge nurse was not a factor affecting stress during the COVID-19 pandemic, and that the level of stress was higher among head nurses. When considering the division of duties in the hospital, charge nurses are responsible for both administrative work and direct nursing care, while head nurses mainly perform administrative work [21]. Since Keimyung University Dongsan Hospital was designated for COVID-19 patients three days after the massive increase in COVID-19 cases and had to be set up urgently, the charge nurses may have been under immense stress because they had to do administrative and nursing work simultaneously in an unfamiliar situation. Therefore, when numerous changes occur in the workload of nurses—both administrative and nursing care—during a pandemic, charge nurses may experience a high level of stress. A solution for this stress is for the administrative work and direct nursing care to be shared among all nurses to lighten the load of those on the front lines.

Front-line nurses, whose own mental health is often at risk, cannot always fulfill their roles of ensuring the care and safety of patients in a pandemic. In other countries (for example, Italy), the response of medical staff to the COVID-19 pandemic is a legal matter [23]. In contrast, in Korea, nurses at the forefront of the COVID-19 pandemic have been regarded as heroes. However, their mental health suffered. Based on the results of this study, long-term measures are necessary for the recovery and prevention of nurses’ mental health deterioration; in addition, a management program focusing on stigma, women, and middle managers is required. Specifically, it is necessary to provide a mental health management program focusing on women and middle managers, alongside legal and financial support from the state and local communities, and counseling to enable front-line nurses to cope with the stigma they face during disease outbreaks. However, there may be insufficient resources to implement such programs in the event of an infectious disease pandemic. Therefore, providing remote support, such as psychiatric teleconsultation, videoconferencing, and telehealth for cognitive behavioral therapy [24] is essential. This study is significant because it is the first in South Korea to investigate the mental health status of nurses at a COVID-19-specialized hospital. However, this study has some limitations. First, these research results should be generalized with caution as this study was conducted among nurses at a single COVID-19-specialized hospital. Second, nurses’ family members’ experiences of COVID-19 infection, loss of family members/friends due to COVID-19, and alcohol and caffeine intake were not included in the data collection. Therefore, it is necessary to investigate these factors in future studies. Third, this study involved a cross-sectional survey; at the time of the data collection, the influence of global interest, support for COVID-19 hospitals, and degree of state control affecting nurses’ mental health statuses cannot be ruled out. Thus, a follow-up study is needed to substantiate the results. Fourth, nurses’ fear of COVID-19 infection and their experiences of COVID-19 infection were not investigated. Therefore, these variables should be included in future studies to ascertain their effect on the mental health status of nurses.

## 5. Conclusions

This study aimed to investigate the mental health status (depression, anxiety, and stress) of nurses who provided patient care at the first COVID-19-specialized hospital in South Korea and to identify the factors affecting their mental health. Of the participating nurses, 30.6% had moderate or higher depression, 41% had moderate or higher anxiety, and 19.4% had moderate or higher stress. The results also revealed that the higher the stigma, the higher the depression, anxiety, and stress. Depression was higher in female nurses than in male nurses, and stress was higher in charge nurses than in nurses in other positions. Based on these results, it is necessary to focus on the mental health status of nurses during pandemics and implement a management program that considers these influencing factors.

## Figures and Tables

**Table 1:** Mental health status of participants (*n* = 180).

Variables	Categories	*n* (%)	M ± SD	Range
Depression	Normal (≤9)	111 (61.7)	-	-
	Mild (10−13)	14 (7.8)	-	-
	Moderate to severe (≥14)	55 (30.6)	-	-
	Total		9.32 ± 8.70	0−34
Anxiety	Normal (≤7)	96 (53.3)	-	-
	Mild (8−9)	10 (5.6)	-	-
	Moderate to severe (≥10)	74 (41.1)	-	-
	Total		8.70 ± 8.42	0–32
Stress	Normal (≤14)	126 (70.0)	-	-
	Mild (15–18)	19 (10.6)	-	-
	Moderate to severe (≥19)	35 (19.4)	-	-
	Total		10.84 ± 9.57	0–40

**Table 2:** Comparison of demographic data and levels of depression, anxiety, stress, and stigma (*n* = 180).

Variables	Categories	*n* (%) or M ± SD	Depression		Anxiety		Stress	
			M ± SD or n (%)	z/t/F/r (*p*)	M ± SD or n (%)	t/F (*p*)	M ± SD or n (%)	t/F (*p*)
Age (year)	≧39	147 (81.7)	8.34 ± 8.08	−3.29 (0.001)	7.77 ± 7.93	−3.21 (0.002)	9.67 ± 9.11	−3.58 (0.000)
	40≥	33 (18.3)	13.70 ± 10.00		12.85 ± 9.40		16.06 ± 9.99	
Gender	Men	10 (5.6)	2.00 ± 5.66	−3.74 ^2^ (<0.001)	2.40 ± 5.56	−3.10 ^2^ (0.002)	4.00 ± 5.42	−2.68 ^2^ (0.007)
	Women	170 (94.4)	9.75 ± 8.66		9.07 ± 8.42		11.25 ± 9.62	
Marital status	Single	49 (27.2)	11.18 ± 8.72	1.77	10.82 ± 8.55	2.08	13.35 ± 9.62	2.17
	Married	131 (72.8)	8.63 ± 8.61	(0.079)	7.91 ± 8.26	(0.039)	9.91 ± 9.42	(0.32)
Living with children	Yes	38 (21.1)	13.00 ± 9.20	−3.19 (0.001)	12.42 ± 8.90	−3.14 (0.002)	15.63 ± 9.96	−3.53 (<0.001)
	No	142 (78.9)	8.34 ± 8.31		7.70 ± 8.03		9.56 ± 9.08	
Change of residence	Yes	70 (38.9)	8.83 ± 7.70	−0.61	8.89 ± 8.09	0.235	10.94 ± 9.34	0.110
	No	110 (61.1)	9.64 ± 9.29	(0.545)	8.58 ± 8.65	(0.814)	10.78 ± 9.75	(0.913)
Position	nurse (a)	156 (86.7)	8.51 ± 8.11	6.06 (0.003) a = c < b	8.08 ± 7.98	4.97 (0.008) a = c < b	9.90 ± 9.05	8.19 (0.000) a = c < b
	Charge nurse (b)	13 (7.2)	16.46 ± 10.30		15.54 ± 9.67		20.46 ± 11.14	
	Head nurse or above (c)	11 (6.1)	12.36 ± 10.87		9.45 ± 10.12		12.91 ± 9.05	
Educational level	Bachelors	155 (86.1)	10.27 ± 9.32	2.10 (0.126)	8.15 ± 8.08	2.79 (0.064)	8.85 ± 8.36	1.91 (0.151)
	Masters	23 (12.8)	14.61 ± 10.88		12.52 ± 9.86		12.61 ± 10.44	
	Doctoral	2 (1.1)	12.00 ± 5.66		7.00 ± 9.89		8.00 ± 8.49	
Department	COVID-19 ward	95 (52.8)	7.94 ± 7.91	3.12 (0.056)	7.79 ± 8.18	1.43 (0.242)	9.62 ± 9.36	3.23 (0.046)
	ICU ^1^	72 (40.0)	10.47 ± 9.101		9.44 ± 8.42		1.44 ± 9.23	
	Infection control administration	13 (7.2)	13.08 ± 10.41		11.23 ± 9.88		16.46 ± 11.35	
Work experience of infectious diseases	Yes	13 (7.2)	10.15 ± 8.58	−0.68 ^2^ (0.494)	10.62 ± 9.18	−0.87 ^2^ (0.384)	13.69 ± 9.12	−1.52 ^2^ (0.129)
	No	167 (92.8)	9.26 ± 8.72		8.55 ± 8.37		10.62 ± 9.60	
Stigma		38.94 ± 12.37		0.42 (<0.001)		0.46 (<0.001)		0.44 (<0.001)

^1^ ICU: Intensive Care Unit. ^2^ Mann–Whitney U test.

**Table 3:** Factors influencing depression (*n* = 180).

Variables	B	β	t	*p*
Stigma	0.29	0.41	5.97	<0.001
Gender (Women)	5.42	0.14	2.11	0.036
F = 25.47, *p* < 0.001, Adj. R^2^ = 0.19				

Durbin–Watson: 2.10.

**Table 4:** Factors influencing anxiety (*n* = 180).

Variables	B	β	t	*p*
Stigma	0.31	0.46	6.88	<0.001
F = 47.30, *p* < 0.001, Adj. R^2^ = 0.21				

Durbin–Watson: 2.08.

**Table 5:** Factors influencing stress (*n* = 180).

Variables	B	β	t	*p*
Stigma	0.31	0.40	5.76	<0.001
Job position (Charge nurse)	6.33	0.17	2.49	0.014
F = 25.59, *p* < 0.001, Adj. R^2^ = 0.22				

Durbin–Watson: 2.12.

## Data Availability

The data presented in this study are available on request from the corresponding author. However, to maintain confidentiality, the data are not publicly available.

## References

[1] Gross J.V., Mohren J., Erren T.C. (2021). COVID-19 and healthcare workers: A rapid systematic review into risks and preventive measures. BMJ.

[2] Nioi M., Napoli P.E., Lobina J., Fossarello M., d’Aloja E. (2020). COVID-19 and Italian healthcare workers from the initial sacrifice to the MRNA vaccine: Pandemic chrono-history, Epidemiological data, ethical dilemmas, and future challenges. Front. Public Health.

[3] Pappa S., Ntella V., Giannakas T., Giannakoulis V.G., Papoutsi E., Katsaounou P. (2020). Prevalence of depression, anxiety, and insomnia among healthcare workers during the COVID-19 pandemic: A systematic review and meta-analysis. Brain Behav. Immun..

[4] Muller A.E., Hafstad E.V., Himmels J.P.W., Smedslund G., Flottorp S., Stensland S.Ø., Stroobants S., Van De Velde S., Vist G.E. (2020). The mental health impact of the Covid-19 pandemic on healthcare workers, and interventions to help them: A rapid systematic review. Psychiatry Res..

[5] Maunder R., Hunter J., Vincent L., Bennett J., Peladeau N., Leszcz M., Sadavoy J., Verhaeghe L.M., Steinberg R., Mazzulli T. (2003). The immediate psychological and occupational impact of the 2003 SARS outbreak in a teaching hospital. CMAJ.

[6] Henry J.D., Crawford J.R. (2005). The short-form version of the depression anxiety stress scales (DASS-21): Construct validity and normative data in a large non-clinical sample. Br. J. Clin. Psychol..

[7] Lee K.W., Kim S.J., Park J.B., Lee K.J. (2011). Relationship between depression anxiety stress scale (DASS) and urinary hydroxyproline and proline concentrations in hospital workers. J. Prev. Med. Public Health.

[8] Defleur M.L., Goffman E. (1964). Stigma: Notes on the management of spoiled identity. Soc. Forces.

[9] Park J.S., Lee E.H., Park N.R., Choi Y.H. (2018). Mental health of nurses working at a government-designated hospital during a MERS-CoV outbreak: A cross-sectional study. Arch. Psychiatr. Nurs..

[10] Sun H., Wang S., Wang W., Han G., Liu Z., Wu Q., Pang X. (2021). Correlation between emotional intelligence and negative emotions of front-line nurses during the COVID-19 epidemic: A cross-sectional study. J. Clin. Nurs..

[11] Sampaio F., Sequeira C., Teixeira L. (2020). Nurses’ mental health during the Covid-19 outbreak: A cross-sectional study. J. Occup. Environ. Med..

[12] Park J.-H., Jung Y.-M., Lee H.-J., Seo J.-Y. (2018). Prevalence and factors related to irritable bowel syndrome in university students. J. Korean Acad. Fundam. Nurs..

[13] Sampaio F., Sequeira C., Teixeira L. (2021). Impact of COVID-19 outbreak on nurses’ mental health: A prospective cohort study. Environ. Res..

[14] Lee S.M., Kang W.S., Cho A.-R., Kim T., Park J.K. (2018). Psychological impact of the 2015 MERS outbreak on hospital workers and quarantined hemodialysis patients. Compr. Psychiatry.

[15] Uvais N.A., Shihabudheen P., Hafi N.A.B. (2020). Perceived stress and stigma among doctors working in COVID-19–designated hospitals in India. Prim. Care Companion CNS Disord..

[16] Uvais N.A., Aziz F., Hafeeq B. (2020). COVID-19-related stigma and perceived stress among dialysis staff. J. Nephrol..

[17] Badrfam R., Zandifar A. (2020). Stigma over COVID-19; new conception beyond individual sense. Arch. Med Res..

[18] Bagcchi S. (2020). Stigma during the COVID-19 pandemic. Lancet Infect. Dis..

[19] Chen S., Xia M., Wen W., Cui L., Yang W., Liu S., Fan J., Yue H., Tang S., Tang B. (2020). Mental health status and coping strategy of medical workers in China during the COVID-19 outbreak. MedRxiv.

[20] Wang C., Pan R., Wan X., Tan Y., Xu L., Ho C.S., Ho R.C. (2020). Immediate psychological responses and associated factors during the initial stage of the 2019 coronavirus disease (COVID-19) epidemic among the general population in China. Int. J. Environ. Res. Public Health.

[21] Lee B.S., Ko H.J., Kwon Y.S., Kim J.N., Park Y.S., Park J.S. (2001). Activities of head nurses and charge nurses in general hospital. Keimyung J. Nurs. Sci..

[22] McCallin A., Frankson C. (2010). The role of the charge nurse manager: A descriptive exploratory study. J. Nurs. Manag..

[23] D’Aloja E., Finco G., Demontis R., Napoli P.E., Fossarello M., Nioi M. (2020). COVID-19 and medical liability: Italy denies the shield to its heroes. EClinicalMedicine.

[24] Dent L., Peters A., Kerr P.L., Mochari-Greenberger H., Pande R.L. (2018). Using telehealth to implement cognitive-behavioral therapy. Psychiatr. Serv..

